# Effects of different levels of dietary crude protein on growth performance, blood profiles, diarrhea incidence, nutrient digestibility, and odor emission in weaning pigs

**DOI:** 10.5713/ab.22.0440

**Published:** 2023-02-27

**Authors:** Hongjun Kim, Haewon Shin, Yoo Yong Kim

**Affiliations:** 1 Department of Agricultural Biotechnology and Research Institute of Agriculture and Life Sciences, Seoul National University, Seoul 08826, Korea

**Keywords:** Diarrhea Incidence, Dietary Crude Protein Level, Growth Performance, Nutrient Digestibility, Odor Emission

## Abstract

**Objective:**

This experiment was conducted to evaluate the effects of different levels of dietary crude protein (CP) on growth performance, blood profiles, diarrhea incidence, nutrient digestibility, and odor emission in weaning pigs.

**Methods:**

A total of 240 weaning ([Yorkshire×Landrace]×Duroc) pigs (8.25±0.050 kg body weight [BW]) were assigned to six treatments based on sex and initial BW, with five replicates of eight pigs per pen in a randomized complete block design. Experimental diets with different crude protein levels for early and late weaning phases were as follows: i) CP16, corn-soybean-based diet containing 16%/15% CP; ii) CP17, corn-soybean-based diet containing 17%/16% CP; iii) CP18, corn-soybean-based diet containing 18%/17% CP; iv) CP19, corn-soybean-based diet containing 19%/18% CP; v) CP20, corn-soybean-based diet containing 20%/19% CP; and vi) CP21, corn-soybean-based diet containing 21%/20% CP.

**Results:**

In the early weaning period, average daily feed intake increased when the dietary CP level decreased (linear, p<0.05). During the entire experimental period, average daily gain and the gain to feed ratio decreased when the dietary CP level increased (linear, p< 0.01). Additionally, a decrease in dietary CP level resulted in a linear increase in final BW (linear, p<0.05). In the early and late weaning periods, blood urea nitrogen (BUN) decreased when the dietary CP level decreased (linear, p<0.01). There were no significant differences in creatinine, glucose, total protein, triglyceride or insulin-like factor-1 levels over the experimental period. The concentrations of immunoglobulin A (IgA) and IgG were not significantly affected by dietary CP levels during the experimental period. In the early weaning period, fecal and urine N decreased when the dietary CP level decreased (linear, p<0.01). No differences in nutrient digestibility among the treatments during the early weaning period were found. Throughout the whole experimental period, when the dietary CP level decreased in the weaning pig diet, the diarrhea incidence decreased linearly (linear, p<0.01). Throughout the whole experimental period, when the dietary CP level decreased in the weaning pig diet, ammonia, amines and hydrogen sulfide decreased linearly (linear, p<0.01).

**Conclusion:**

Reducing dietary CP could decrease diarrhea incidence, the concentration of BUN in serum and odor emission in manure. Furthermore, it could improve N excretion in feces and urine and growth performance in weaning pigs.

## INTRODUCTION

From the past to the present, a high-protein diet is mainly supplied to increase the productivity of swine farms. When weaning pigs are fed a high-protein diet, they increase the amount of undigested protein that cannot be fermented in the large intestine, thereby promoting pathogenic bacterial growth in the gastrointestinal tract [1,2]. In addition, the incidence of diarrhea increases after weaning when a high-protein diet is fed to weaning pigs, and the incidence of diarrhea decreases when a low-protein diet is provided [3–5]. Additionally, bacterial fermentation of undigested protein produces volatile fatty acid (VFA) and potentially toxic substances such as ammonia and amines that can reduce the growth of weaned piglets [6]. To solve the problem of diarrhea in weaning pigs, the weaning pig diet was supplemented with zinc oxide for pharmacological purposes in the past. However, the use of zinc oxide for pharmacological purposes is being reduced worldwide.

A total of 137 million tons of nitrogen from livestock raised worldwide has been emitted [7]. In addition, there have been severe environmental problems caused by nitrogen in the swine industry, and many efforts have been made to reduce nitrogen emissions [8]. In the past, studies have mainly been conducted on fundamental factors related to animal physiological properties and genetics [9]. However, studies are currently being conducted to assess reductions in protein levels in the diet as a means to reduce the release of nitrogen emissions through feces and urine [10]. Reducing dietary crude protein (CP) with sufficient amino acid supplementation in a weaning pig diet did not show any adverse effects on the growth performance of pigs but reduced nitrogen retention [11]. Philippe et al [12] and Le et al [13] reported that reducing dietary CP with adequate amino acid supplementation in a weaning pig diet significantly decreased the emission of ammonia while retaining the standard growth performance of weaning pigs.

Thus, it was hypothesized that effects of low crude protein could improve diarrhea incidence and nutrient utilization, leading to an improvement of growth performance in weaning pigs. Therefore, this experiment was designed to evaluate the effect of different levels of dietary crude protein on growth performance, blood profiles, diarrhea incidence, nutrient digestibility, and odor emission in weaning pigs.

## MATERIALS AND METHODS

### Experimental animals and management

All experimental procedures involving animals were conducted following the Animal Experimental Guidelines provided by the Seoul National University Institutional Animal Care and Use Committee (SNUIACUC; SNU-210811-6). A total of 240 weaning ([Yorkshire×Landrace]× Duroc) pigs (8.25±0.050 kg body weight [BW]) were allotted to one of six treatments based on sex and initial BW, with five replicates of eight pigs per pen in a randomized complete block design. The pigs were allotted randomly to their respective treatments using an experimental animal allotment program [14]. The pens were contained within a concrete-floored, environmentally controlled facility (1.54×1.96 m) equipped with a feeder and water nipples at the Seoul National University Farm. The experimental period was 6 weeks and consisted of phase 1 (the first 3 weeks) and phase 2 (weeks 4 to 6).

### Experimental design and diet

Experimental diets with different crude protein levels for early and late weaning phases were as follows: i) CP16, corn-soybean-based diet containing 16%/15% CP; ii) CP17, corn-soybean-based diet containing 17%/16% CP; iii) CP18, corn-soybean-based diet containing 18%/17% CP; iv) CP19, corn-soybean-based diet containing 19%/18% CP; v) CP20, corn-soybean-based diet containing 20%/19% CP; and vi) CP21, corn-soybean-based diet containing 21%/20% CP. The experimental diets were formulated for weaning phases 1 and 2. All experimental early weaning diets were formulated to contain 3,400 kcal of metabolizable energy (ME)/kg, 1.35% total lysine, 0.39% total methionine, 0.83% total threonine, 0.24% total tryptophan, 0.80% calcium and 0.65% total phosphorus. All experimental late weaning diets were formulated to contain 3,350 kcal of ME/kg, 1.23% total lysine, 0.36% total methionine, 0.76% total threonine, 0.22% total tryptophan, 0.70% calcium, and 0.60% total phosphorus. All other nutrients were formulated to meet or exceed the NRC [15] requirements. The formula and chemical composition of the experimental diets are presented in Tables 1 and 2.

### Growth performance

The BW and feed intake data were collected at the end of each phase to calculate average daily gain (ADG), average daily feed intake (ADFI), and the gain to feed (G:F) ratio. In addition, the amount of feed eaten by all piglets was recorded each day, and waste feed in the feeder was recorded at the end of each phase. All pigs were housed in slotted plastic floor pens equipped with a feeder and a nipple waterer and allowed *ad libitum* access to feed and water throughout the whole experimental period. The temperature in the experimental house was maintained at 30°C in the first week, decreased by 1°C every week, and was 24°C in the last week.

### Blood sampling and analyses

Blood samples were taken from the jugular vein of six pigs with nearly average BWs in each treatment after 3 h of fasting to measure blood urea nitrogen (BUN), creatinine, glucose, total protein, triglycerides (TG), IgA, IgG, and insulin-like growth factor-1 (IGF-1) when BW was recorded. The blood samples were centrifuged for 15 min at 3,000 rpm and 4°C (centrifuge 5810R; Eppendorf, Hamburg, Germany). The sera were transferred to 1.5 mL plastic tubes (serum tubes, BD vacutainer SST II advance; Becton-Dickinson, London, UK) and stored at −20°C until analysis. Enzyme-linked immunosorbent assays (ELISAs) were performed using an absorbance microplate reader (VERSA Max) to determine the concentrations of insulin (Porcine Insulin ELISA Kit), IgA (Pig IgA ELISA Kit), IgG (Pig IgG ELISA Kit), and IGF-1 (pig insulin-like growth factor-1, IGF-1 ELISA Kit). BUN was analyzed using a Cobas 6000 by a kinetic/photometric method. Creatinine and total protein were analyzed using a Cobas 6000 by a colorimetric method. Glucose was analyzed using a Cobas 6000 by an enzymatic UV/hexokinase method. Triglycerides were analyzed using a Cobas 6000 by an enzymatic colorimetric method.

### Nutrient digestibility

Eighteen crossbred barrows (mean, 8.41±0.371 kg BW) were allotted to individual metabolic crates (40×80×90 cm) in a completely randomized design with three replicates to evaluate nutrient digestibility and nitrogen retention. The total collection method was used to determine apparent total tract digestibility. A 5-day collection period followed the 5-day adaptation period. To identify the first and last collection days, 8 g of iron oxide and chromium oxide were added to the first and last day experimental diets as selection markers. All pigs were fed 256 g of the experimental diet twice daily at 7:00 and 19:00 during the experimental period, which was three times the maintenance energy, and water was provided *ad libitum*. Feces were collected using the total collection method, and urine was collected daily in a plastic container. Feed intake, feces, and urine were recorded every day. The feces and urine samples were stored at −20°C until analysis. The excreta were pooled and dried in a forced-air drying oven at 60°C for 72 h and then ground into 1-mm particles with a Wiley mill for chemical analysis. A sulfuric acid solution, which collected the ammonia in urine by a chemical reaction, was titrated from a 99% sulfuric acid solution to a 10% sulfuric acid solution and was added to a 50-mL plastic case. Glass wool was put into a funnel that was attached to the plastic case to prevent impurities from entering. The urine was then passed through glass wool into the plastic case and diluted with 2 L of water. The diluted urine was collected in a 50-mL conical tube and stored at −20°C before analysis. Moisture, CP, crude fat, and crude ash were analyzed by AOAC (1995) [16] methods for chemical composition analysis of feed, feces, and urine.

### Incidence of diarrhea

The diarrhea incidence was determined at 8:00 am for 35 days. Data were recorded by pen during phases 1 and 2. The incidence of diarrhea was scored on a 4-point scale according to the condition of the feces and diarrhea (0 = normal feces; 1 = moist feces; 2 = mild diarrhea; 3 = severe and watery diarrhea). Slightly wet feces on the rump area was designated as contaminated. After recording these data, the watery diarrhea was cleaned away.

### Odor gas emission

For the odor gas estimation, 500 g of fresh feces and 400 g of urine were mixed following the methods of Kim et al [17]. Mixtures of fecal and urine were fermented at room temperature of 35°C for 72 hours. The odor-causing materials (amines, ammonia and hydrogen sulfide) were analyzed every 24 hours for 7 days with a gas detector (GV-110S; Gastec, Ayase, Japan) and tube (namely, an amine detector tube [180, 5 to 100 ppm], ammonia detector tube [3L, 0.5 to 78 ppm] and hydrogen sulfide detector tube [4 LT, 0.05 to 4 ppm]).

### Statistical and chemical analyses

Experimental diet and excreta were analyzed for contents of dry matter (procedure 967.03; AOAC, 1995), ash (procedure 923.03; AOAC, 1995), N by using the Kjeldahl procedure with a Kjeltec instrument (Kjeltec 2200; Foss Tecator, Höganäs, Sweden) and CP content (Nitrogen×6.25; procedure 981.10; AOAC, 1995). All of the collected data were subjected to least squares mean comparisons and were evaluated with the general linear model procedure of SAS [18]. Orthogonal polynomial contrast was used to determine linear and quadratic effects according to increases in the dietary crude protein levels of the weaning diets. In the case of growth performance, a pen was considered an experimental unit, while an individual piglet was used as a unit to analyze nutrient digestibility, blood profile, immunity, incidence of diarrhea and odor emission. Differences among means were declared significant at p<0.05 and highly significant at p<0.01, and the determination of tendency for all analyses was p≥0.05 and p<0.10.

## RESULTS

### Growth performance

The effects of different levels of dietary CP on growth performance are presented in Table 5. In the early weaning period, the ADFI increased when the dietary CP level decreased (linear, p<0.05). During the entire experimental period, ADG and the G:F ratio decreased when the dietary CP level increased (linear, p<0.01). Additionally, a decrease in dietary CP level resulted in a linear increase in final BW (linear, p<0.05).

### Blood profiles

The effects of different levels of dietary CP on blood profiles are presented in Table 6. In the early and late weaning periods, BUN decreased when the dietary CP level decreased (linear, p<0.01). There were no significant differences in creatinine, glucose, total protein, triglyceride or IGF-1 levels over the experimental period.

### Immune response

The effects of different levels of dietary CP on the immune response are presented in Table 7. The concentrations of IgA and IgG were not significantly affected by dietary CP levels during the whole experimental period.

### Nutrient digestibility

The effects of different levels of dietary CP on nutrient digestibility and nitrogen retention are presented in Table 8. In the early weaning period, fecal and urine N decreased when the dietary CP level decreased (linear, p<0.01). No differences were found in nutrient digestibility among the treatments during the early weaning period.

### Diarrhea incidence

The effects of different levels of dietary CP on the incidence of diarrhea are presented in Table 9. Throughout the whole experimental period, when the dietary CP level decreased in the weaning pig diet, the diarrhea incidence decreased linearly (linear, p<0.01).

### Odor emission

The effects of different levels of dietary CP on odor emission are presented in Table 10. Throughout the whole experimental period, when the dietary CP level decreased in the weaning pig diet, ammonia, amines and hydrogen sulfide decreased linearly (linear, p<0.01).

## DISCUSSION

The effect of dietary CP level on the growth performance of weaning pigs is an issue of debate between researchers. Luo et al [19] reported a significant reduction in ADG and ADFI for weaning pig diets containing dietary CP levels of 14%. Nyachoti et al [20] reported that decreasing the dietary CP level from 23% to 17% in a weaning pig diet decreased the final BW. In addition, ADG, ADFI, and the G:F ratio decreased in the treatment with 17% dietary CP. Yue and Qiao [21] reported that ADG and the G:F ratio decreased when the dietary CP level decreased from 21.2% to 17.2%. Cui et al [22] reported that reducing dietary CP levels from 18.8% to 17.2% had no influence on ADG and the G:F ratio during the experimental period. Heo et al [5] reported that decreasing the dietary CP level from 25.6% to 17.5% in a weaning pig diet had no effect on ADG, ADFI, or the G:F ratio. Peng et al [23] reported that decreasing the dietary CP level from 20% to 13.9% in the weaning pig diet decreased BW and ADG linearly, while the ADFI showed no significant difference among the treatments.

In this study, decreasing the dietary CP level from 21% to 16% while maintaining ideal ratios for other essential amino acids, such as lysine, methionine, threonine and tryptophan, increased the final BW, ADG, and G:F ratio of weaning pigs throughout the entire experimental period. A similar result for ADG and the G:F ratio was reported by Zhou et al [24] and Yue and Qiao [21]. It has been shown that feeding a low CP diet supplemented with amino acids reduces the incidence of diarrhea and improves the health of the gastrointestinal tract compared to a high CP diet [25,26]. These results demonstrated that reducing dietary CP levels from 21% to 16% improved the gut health of weaning pigs and reduced the incidence of diarrhea. Therefore, it improved the growth performance of weaning pigs during the experimental period. In addition, this study suggested that a low CP diet is recommended during the weaning period.

Peng et al [23] reported that plasma urea nitrogen decreased linearly as the level of crude protein decreased (13.9%/15.3%/17.16%/20%). Nyachoti et al [20] reported that plasma urea nitrogen decreased linearly as the level of crude protein decreased (17%/19%/21%/23%). Heo et al [5] reported a decrease in plasma urea nitrogen of urea in the treatment with a 17.5% crude protein level when 17.5% or 23.6% of crude protein was added to the feed. Heo et al [25] reported that when crude protein was added at 17.3% or 24.3% in the feed, the plasma nitrogen of crude protein was low in the treatment with 17.3% crude protein. Yu et al [27] reported that when different percentages of crude protein (14%/17%/20%) was added in the diet, plasma urea nitrogen (PUN) decreased linearly as the level of crude protein addition decreased.

Blood creatinine is an indicator used to calculate muscle content in the body, and blood creatinine concentration is known to be positively correlated with muscle mass in the body [28]. Triglycerides are an indicator of fat metabolism in the body. It is known that when an amino acid imbalance situation occurs in the feed, amino acids that are not used for protein synthesis turn into TG and increase back fat [29]. IGF-1 is activated by the stimulation of growth hormones and is related to the development and differentiation of tissue. IGF-1 is affected by the nutritional status of animals. The serum total protein level is an indicator of protein status in pigs. Furthermore, it indicates that the protein state of pigs improves as the serum total protein level increases [30]. The usual range of total protein levels in serum is known to be 4.41 to 6.80 mg/dL [31]. Therefore, in this experiment, the level of dietary CP in the weaning diet did not have a harmful effect on the total serum protein level. In general, BUN is the final product of proteolysis. It is known that absorption of excess protein increases the BUN concentration and that the BUN concentration decreases as nitrogen accumulation and protein synthesis increase in muscles [32]. Blood urea nitrogen, which is the end-product of protein metabolism, is an index of protein utilization and affects the balance of amino acid and nitrogen intake [33,34]. In this study, weaning pigs fed a low CP diet had a lower BUN concentration than weaning pigs fed a high CP diet during the weaning period. These results were in agreement with the results of Peng et al [23] and Heo et al [25], which demonstrated that decreasing the dietary CP level in a weaning pig diet decreased the BUN concentration. The present study showed that the concentration of BUN decreased linearly as the level of crude protein decreased by 16%, which is considered to be the effect of improving the nitrogen utilization. It is determined that the improvement of the nitrogen digestibility results in a decrease in the BUN concentration in the blood by increasing the nitrogen utilization, and further reduces the emission of surplus nutrients to reduce organic matter in feces and urine.

Immunoglobulin consists of IgG, IgA, IgD, IgE, and IgM, with IgG and IgA accounting for 70 and 20 percent of total globulin, respectively. In general, antibodies of the IgG subtype are present at a high level in the serum and play a role in preventing the invasion of harmful infections and bacteria during the weaning period. IgA is also involved in similar infection prevention but is known to play a role in the mucosa as a secretory antibody. The role of preventing damage to intestinal villi is mainly performed by antibodies, such as IgA and IgG [35]. Weaning pig immunity stems from breast milk absorption [36], and it takes 35 days to synthesize immune proteins in the body [37]. IgG represents body fluid immunity, and IgG absorbed through the placenta is involved in the immune system immediately after birth and plays a crucial role in the defense against diarrhea in weaning pigs caused by external pathogens that occur after birth [38]. IgA accounts for the most significant proportion of immunoglobulin in breast milk and carries a memory of pathogens that the sow once encountered. IgA antibodies secreted from breast milk form an immune system in the mucous membrane in areas prone to pathogenic *Escherichia coli* (*E. coli*), such as the digestive, respiratory, and urinary systems, to defend against bacteria that cause infection through the oral cavity until weaning pigs produce antibodies on their own [39].

Lee et al [40] reported that dietary CP levels did not have any deleterious effect on the concentrations of IgA and IgG. Kim et al [41] reported that IgG was not affected by dietary CP level. In the results from the current experiment, there were no significant differences among the experimental diets in IgA and IgG. This result is similar to the observation of Lee, who stated that the concentrations of IgA and IgG did not show any significant difference when dietary CP levels were decreased. This is in agreement with the finding of Kim et al [41], who stated that feeding a low CP diet did not affect the concentration of IgG among treatments. The results from the current study showed similar results to those reported by Lee et al [40] and Kim et al [41].

Zhou et al [24] reported that when dietary CP was supplemented at 18.5% or 20% in the weaning pig diet, this resulted in a decrease in N excretion in treatment with dietary CP at 18.5%. Lordelo et al [42] reported that when dietary CP was added at 17% or 20% in a weaning pig diet, N excretion in feces from the treatment with 17% dietary CP was reduced. Bujňák et al [43] reported that when a dietary CP level of 16.7% or 19.7% was added to weaning pig diets, the dietary CP level and N contents in feces and urine tended to be proportional. Ball et al [44] reported that when the dietary CP level decreased from 19.3% to 14.4%, N excretion in feces and urine from animals fed a diet with 19.3% CP increased. Kim et al. reported that when dietary CP was supplemented at 15.1% or 17.3% in the diet, N excretion in feces and urine after feeding with 15% CP decreased.

Pfeiffer et al [45] reported that there was a positive correlation between the CP level in the diet and N excretion in feces and urine. In addition, it was reported that an excess supply of dietary CP leads to a release of a large amount of nitrogen [46]. Providing a diet containing excessive dietary CP to weaning pigs exceeds weaning pigs’ ability to digest crude protein, which causes diarrhea and increases N excretion in feces and urine. Therefore, the highest level of CP in this study showed low growth performance, and the BUN concentration was also the highest. It also had the highest diarrhea incidence during the whole experimental period. These results are consistent with the results of several previous studies [24,42–44,47]. It is believed that the excessive supply of dietary CP exceeds the digestive capacity of weaning pigs, resulting in an increase in N excretion in feces and urine.

Lynegaard et al [2] reported the lowest incidence of diarrhea in the treatment with 15.1% dietary CP when dietary CP in the diet was reduced from 19.1% to 15.1%. Opapeju et al [48] reported that when dietary CP was added at 17.3% or 22.2% in a weaning pig diet, enterotoxigenic *E. coli* K88 was found in the treatment with 17.3% dietary CP, and the diarrhea incidence increased. Heo et al [25] reported that the incidence of diarrhea in treatments with a low CP diet decreased when crude protein was added at 17.3% or 24.3%. Additionally, the *E. coli* level in the feces increased after treatment with a high CP diet. Yue and Qiao [21] reported that the incidence of diarrhea decreased after treatment with a low CP diet when the dietary CP in the weaning pig diet decreased from 21.2% to 17.2%. Park et al [49] reported that the incidence of diarrhea decreased for two weeks after treatment with 22.51% dietary CP when 24.49% or 22.51% crude protein was added to the feed.

Reducing dietary CP levels in the weaning pig diet has been shown to decrease diarrhea incidence because protein fermentation by proteolytic bacteria in the hindgut decreases [5]. Additionally, the number of enterotoxigenic *E. coli* K88 in the digesta in the small intestine and large intestine was reduced with a low CP diet [26]. Similar to the results of several previous studies [5,21,25,26], in this study, N excretion in feces and urine decreased, and the BUN concentration decreased as the level of dietary CP in the diet decreased. Therefore, it is believed that protein utilization was the highest after treatment with the low CP diet, which was also associated with an improved growth performance and lowest incidence of diarrhea.

Reducing the dietary CP in the weaning diet is effective for reducing protein fermentation in the gastrointestinal tract [50]. Undigested dietary proteins and endogenous proteins enter the large intestine, producing toxic metabolites that can impair epithelial integrity and promote intestinal diseases such as the incidence of diarrhea after weaning [51]. Portejoie et al [52] reported that ammonia emissions decreased as the level of dietary CP in the diet decreased (12%/16%/20%). Lee et al [40] reported that ammonia, hydrogen sulfide, and VFA emissions were significantly lower after treatment with 19.6% dietary CP when the weaning pig diet was supplemented with 21.7% or 19.6% dietary CP. Leek et al [53] reported that ammonia emissions linearly decrease as the level of dietary CP increases (13.3%/15.7%/19.0%/20.6%). Le et al [13] found a reduction of 80% in odor emission when dietary CP was decreased from 18% to 12%. The results from the current study showed similar results to those reported by Lee et al [40], Portejoie et al [52], and Leek et al [53]. Therefore, in this study, it is considered that the manure odor emission was reduced because N excretion in feces and urine was reduced in weaning pigs fed a low CP diet.

The limitation of current study is that some amino acid requirements such as histidine, isoleucine and phenylalanine does not meet. If all essential amino acid requirements are met in further study, more meaningful results will be obtained.

## CONCLUSION

These results indicated that reducing dietary CP could decrease diarrhea incidence, the concentration of BUN in serum, and odor emission in manure. Furthermore, it could improve N excretion in feces and urine and growth performance in weaning pigs.

## Figures and Tables

**Table 1:** Formula of the experimental diets during the early weaning phase (weeks 0 to 3)

Ingredient (%)	Treatment^1)^					

	CP16	CP17	CP18	CP19	CP20	CP21
Expanded corn	63.05	60.45	57.84	55.30	52.67	50.11
Soybean meal	2.44	5.25	8.07	10.90	13.70	16.53
Fermented soybean meal	8.00	8.00	8.00	8.00	8.00	8.00
Soy oil	1.07	1.12	1.18	1.21	1.26	1.30
Whey base	5.00	5.00	5.00	5.00	5.00	5.00
Lactose base	10.00	10.00	10.00	10.00	10.00	10.00
Fish meal	4.00	4.00	4.00	4.00	4.00	4.00
Blood plasma	2.00	2.00	2.00	2.00	2.00	2.00
L-Lysine sulfate, 55%	0.98	0.84	0.70	0.56	0.43	0.29
DL-Met, 98%	0.15	0.13	0.12	0.10	0.09	0.07
L-Threonine, 98.5%	0.23	0.19	0.14	0.09	0.05	0.00
L-Tryptophan, 99%	0.08	0.07	0.05	0.03	0.02	0.00
Monocalcium phosphate	1.05	1.00	0.95	0.88	0.85	0.79
Limestone	1.15	1.15	1.15	1.13	1.13	1.11
Vit. Mix^2)^	0.10	0.10	0.10	0.10	0.10	0.10
Min. Mix^3)^	0.10	0.10	0.10	0.10	0.10	0.10
Salt	0.30	0.30	0.30	0.30	0.30	0.30
Zinc oxide	0.30	0.30	0.30	0.30	0.30	0.30
Total	100.00	100.00	100.00	100.00	100.00	100.00

1) CP16, corn-SBM-based diet (early weaning CP 16%/late weaning CP 15%); CP17, corn-SBM-based diet (early weaning CP 17%/late weaning CP 16%; CP18, corn-SBM-based diet (early weaning CP 18%/late weaning CP 17%); CP19, corn-SBM-based diet (early weaning CP 19%/late weaning CP 18%); CP20, corn-SBM-based diet (early weaning CP 20%/late weaning CP 19%); CP21, corn-SBM-based diet (early weaning CP 21%/late weaning CP 20%).

2) Quantities of vitamins provided per kg of complete diet: vitamin A, 8,000 IU; vitamin D_3_, 1,800 IU; vitamin E, 80 IU; vitamin K_3_, 2 mg; riboflavin, 7 mg; calcium pantothenic acid, 25 mg; niacin, 27 mg; D-biotin, 200 μg; vitamin B_12_, 50 μg.

3) Quantities of minerals provided per kg of complete diet: Fe, 150 mg; Cu, 105 mg; Mn, 51 mg; I, 1 mg; Se, 0.3 mg; Zn, 72 mg.

**Table 2:** Chemical compositions of the experimental diets during the early weaning phase (weeks 0 to 3)

Chemical composition^1)^	Treatment^2)^					

	CP16	CP17	CP18	CP19	CP20	CP21
ME (kcal/kg)	3,400.00	3,400.00	3,400.00	3,400.00	3,400.00	3,400.00
Crude protein (%)	16.00	17.00	18.00	19.00	20.00	21.00
SID lysine (%)	1.35	1.35	1.35	1.35	1.35	1.35
SID methionine (%)	0.39	0.39	0.39	0.39	0.39	0.39
SID threonine (%)	0.83	0.83	0.83	0.83	0.83	0.83
SID tryptophan (%)	0.24	0.24	0.24	0.24	0.24	0.24
SID arginine (%)	0.88	0.97	1.07	1.17	1.26	1.36
SID histidine (%)	0.41	0.44	0.47	0.50	0.53	0.57
SID isoleucine (%)	0.63	0.69	0.74	0.80	0.86	0.92
SID leucine (%)	1.42	1.51	1.59	1.67	1.76	1.84
SID phenylalanine (%)	0.74	0.80	0.87	0.93	0.99	1.05
SID valine (%)	0.86	0.92	0.97	1.03	1.09	1.15
SID phenylalanine+tyrosine (%)	1.29	1.40	1.51	1.62	1.73	1.84
Total calcium (%)	0.80	0.80	0.80	0.80	0.80	0.80
STTD phosphorus (%)	0.40	0.40	0.40	0.40	0.40	0.40

ME, metabolizable energy; SID, standardized ileal digestible; STTD, standardized total tract digestible.

1) Calculated value.

2) CP16, corn-SBM-based diet (early weaning CP 16%/late weaning CP 15%); CP17, corn-SBM-based diet (early weaning CP 17%/late weaning CP 16%); CP18, corn-SBM-based diet (early weaning CP 18%/late weaning CP 17%); CP19, corn-SBM-based diet (early weaning CP 19%/late weaning CP 18%); CP20, corn-SBM-based diet (early weaning CP 20%/late weaning CP 19%); CP21, corn-SBM-based diet (early weaning CP 21%/late weaning CP 20%).

**Table 3:** Formula of the experimental diets during the late weaning phase (weeks 3 to 6)

Ingredient (%)	Treatment^1)^					

	CP16	CP17	CP18	CP19	CP20	CP21
Expanded corn	72.71	70.13	67.57	64.96	62.36	59.79
Soybean meal	3.18	6.00	8.82	11.65	14.48	17.30
Fermented soybean meal	8.00	8.00	8.00	8.00	8.00	8.00
Soy oil	0.35	0.40	0.43	0.48	0.53	0.57
Whey base	2.50	2.50	2.50	2.50	2.50	2.50
Lactose base	5.00	5.00	5.00	5.00	5.00	5.00
Fish meal	4.00	4.00	4.00	4.00	4.00	4.00
L-Lysine sulfate, 55%	0.97	0.83	0.70	0.56	0.41	0.28
DL-Met, 98%	0.12	0.10	0.09	0.07	0.06	0.04
L-Threonine, 98.5%	0.23	0.19	0.14	0.09	0.05	0.00
L-Tryptophan, 99%	0.09	0.07	0.05	0.04	0.02	0.00
Monocalcium phosphate	1.10	1.03	0.95	0.92	0.85	0.80
Limestone	0.95	0.95	0.95	0.93	0.94	0.92
Vit. Mix^2)^	0.10	0.10	0.10	0.10	0.10	0.10
Min. Mix^3)^	0.10	0.10	0.10	0.10	0.10	0.10
Salt	0.30	0.30	0.30	0.30	0.30	0.30
Zinc oxide	0.30	0.30	0.30	0.30	0.30	0.30
Total	100.00	100.00	100.00	100.00	100.00	100.00

1) CP16, corn-SBM-based diet (early weaning CP 16%/late weaning CP 15%); CP17, corn-SBM-based diet (early weaning CP 17%/late weaning CP 16%); CP18, corn-SBM-based diet (early weaning CP 18%/late weaning CP 17%); CP19, corn-SBM-based diet (early weaning CP 19%/late weaning CP 18%); CP20, corn-SBM-based diet (early weaning CP 20%/late weaning CP 19%); CP21, corn-SBM-based diet (early weaning CP 21%/late weaning CP 20%).

2) Quantities of vitamins provided per kg of complete diet: vitamin A, 8,000 IU; vitamin D_3_, 1,800 IU; vitamin E, 80 IU; vitamin K_3_, 2 mg; riboflavin, 7 mg; calcium pantothenic acid, 25 mg; niacin, 27 mg; D-biotin, 200 μg; vitamin B_12_, 50 μg.

3) Quantities of minerals provided per kg of complete diet: Fe, 150 mg; Cu, 105 mg; Mn, 51 mg; I, 1 mg; Se, 0.3 mg; Zn, 72 mg.

**Table 4:** Chemical compositions of the experimental diets during the early weaning phase (weeks 3 to 6)

Chemical composition^1)^	Treatment^2)^					

	CP16	CP17	CP18	CP19	CP20	CP21
ME (kcal/kg)	3,350.00	3,350.00	3,350.00	3,350.00	3,350.00	3,350.00
Crude protein (%)	15.00	16.00	17.00	18.00	19.00	20.00
SID lysine (%)	1.23	1.23	1.23	1.23	1.23	1.23
SID methionine (%)	0.36	0.36	0.36	0.36	0.36	0.36
SID threonine (%)	0.76	0.76	0.76	0.76	0.76	0.76
SID tryptophan (%)	0.22	0.22	0.22	0.22	0.22	0.22
SID arginine (%)	0.85	0.94	1.04	1.14	1.23	1.33
SID histidine (%)	0.39	0.42	0.45	0.48	0.51	0.54
SID isoleucine (%)	0.60	0.66	0.72	0.78	0.84	0.90
SID leucine (%)	1.37	1.45	1.54	1.62	1.71	1.79
SID phenylalanine (%)	0.70	0.77	0.83	0.89	0.96	1.02
SID valine (%)	0.79	0.85	0.91	0.97	1.02	1.08
SID phenylalanine+tyrosine (%)	1.20	1.32	1.43	1.54	1.65	1.76
Total calcium (%)	0.70	0.70	0.70	0.70	0.70	0.70
STTD phosphorus (%)	0.33	0.33	0.33	0.33	0.33	0.33

ME, metabolizable energy; SID, standardized ileal digestible; STTD, standardized total tract digestible.

1) Calculated value.

2) CP16, corn-SBM-based diet (early weaning CP 16%/late weaning CP 15%); CP17, corn-SBM-based diet (early weaning CP 17%/late weaning CP 16%); CP18, corn-SBM-based diet (early weaning CP 18%/late weaning CP 17%); CP19, corn-SBM-based diet (early weaning CP 19%/late weaning CP 18%); CP20, corn-SBM-based diet (early weaning CP 20%/late weaning CP 19%); CP21, corn-SBM-based diet (early weaning CP 21%/late weaning CP 20%).

**Table 5:** Effects of different levels of dietary crude protein on growth performance in weaning pigs^1)^

Criteria	Treatment^2)^						SEM	p-value^3)^	
	
	CP16	CP17	CP18	CP19	CP20	CP21		L	Q
Body weight (kg)									
Initial	---------------------------------------------------- 8.30 ----------------------------------------------------								
3 wk	14.80	14.51	14.12	14.52	14.33	13.54	0.323	0.37	0.84
6 wk	26.62	26.33	25.96	25.96	25.52	23.88	0.397	0.04	0.44
ADG (g)									
0 to 3 wk	309.52	295.24	279.25	297.09	286.04	247.74	9.004	0.11	0.66
3 to 6 wk	563.07	562.69	566.25	545.00	532.80	487.82	10.076	0.78	0.29
0 to 6 wk	436.30	428.96	421.65	420.05	409.42	367.91	7.621	0.01	0.26
ADFI (g)									
0 to 3 wk	638.73	582.86	554.16	568.73	562.41	517.43	14.589	0.03	0.66
3 to 6 wk	882.00	924.28	919.67	871.28	888.86	907.09	10.931	0.90	0.90
0 to 6 wk	785.36	778.57	761.91	745.01	750.63	737.26	8.875	0.08	0.79
G:F ratio									
0 to 3 wk	0.484	0.502	0.504	0.521	0.510	0.476	0.007	0.78	0.13
3 to 6 wk	0.641	0.611	0.618	0.625	0.602	0.541	0.606	0.06	0.35
0 to 6 wk	0.557	0.552	0.554	0.563	0.547	0.499	0.545	0.01	0.14

SEM, standard error of the mean; ADG, average daily gain; ADFI, average daily feed intake; G:F, gain to feed.

1) A total of 240 weaning pigs were fed, starting from an average initial body weight of 8.25±0.050 kg to an average final body weight of 25.71±0.397 kg.

2) CP16, corn-SBM-based diet (early weaning CP 16%/late weaning CP 15%); CP17, corn-SBM-based diet (early weaning CP 17%/late weaning CP 16%); CP18, corn-SBM-based diet (early weaning CP 18%/late weaning CP 17%); CP19, corn-SBM-based diet (early weaning CP 19%/late weaning CP 18%); CP20, corn-SBM-based diet (early weaning CP 20%/late weaning CP 19%); CP21, corn-SBM-based diet (early weaning CP 21%/late weaning CP 20%).

3) L, linear; Q, quadratic.

**Table 6:** Effects of different levels of dietary crude protein on blood profiles in weaning pigs^1)^

Criteria	Treatment^2)^						SEM	p-value^3)^	
	
	CP16	CP17	CP18	CP19	CP20	CP21		L	Q
BUN (mg/dL)									
Initial	-------------------------------------------------------- 5.67 --------------------------------------------------------								
3 wk	2.60	4.60	6.20	7.80	8.40	10.6	0.589	<0.01	0.72
6 wk	3.20	6.40	5.00	6.80	9.80	9.00	0.510	<0.01	0.74
Creatinine (mg/dL)									
Initial	-------------------------------------------------------- 0.94 --------------------------------------------------------								
3 wk	0.74	0.71	0.69	0.77	0.62	0.72	0.023	0.55	0.82
6 wk	0.93	0.96	1.05	0.91	0.9	0.87	0.024	0.21	0.18
Glucose (mg/dL)									
Initial	-------------------------------------------------------- 107.50 ------------------------------------------------------								
3 wk	89.83	96.40	90.80	89.40	81.20	91.40	2.056	0.23	0.32
6 wk	97.40	78.20	92.80	81.80	86.80	80.60	2.748	0.22	0.66
Total protein (mg/dL)									
Initial	-------------------------------------------------------- 5.00 --------------------------------------------------------								
3 wk	4.64	4.46	4.76	4.42	4.88	4.60	0.090	0.72	0.92
6 wk	5.22	5.36	4.86	4.88	5.44	5.38	0.097	0.59	0.14
Triglycerides (mg/dL)									
Initial	-------------------------------------------------------- 39.50 --------------------------------------------------------								
3 wk	40.60	30.80	45.80	48.60	45.80	40.80	2.740	0.40	0.46
6 wk	41.40	66.00	35.20	53.80	55.00	59.40	2.682	0.08	0.56
IGF-1 (ng/mL)									
Initial	-------------------------------------------------------- 10.48 --------------------------------------------------------								
3 wk	11.57	9.85	11.64	10.72	12.39	12.59	0.839	0.60	0.72
6 wk	7.08	8.40	8.53	7.27	9.78	7.84	0.415	0.57	0.60

SEM, standard error of the mean; BUN, blood urea nitrogen; IGF-1, insulin-like growth factor.

1) Least squares means of twelve observations per treatment.

2) CP16, corn-SBM-based diet (early weaning CP 16%/late weaning CP 15%); CP17, corn-SBM-based diet (early weaning CP 17%/late weaning CP 16%); CP18, corn-SBM-based diet (early weaning CP 18%/late weaning CP 17%); CP19, corn-SBM-based diet (early weaning CP 19%/late weaning CP 18%); CP20, corn-SBM-based diet (early weaning CP 20%/late weaning CP 19%); CP21, corn-SBM-based diet (early weaning CP 21%/late weaning CP 20%).

3) L, linear; Q, quadratic.

**Table 7:** Effects of different levels of dietary crude protein on immune response in weaning pigs^1)^

Criteria	Treatment^2)^						SEM	p-value^3)^	
	
	CP16	CP17	CP18	CP19	CP20	CP21		L	Q
IgA (mg/mL)									
Initial	---------------------------------------------------------- 0.26 ----------------------------------------------------------								
3 wk	0.66	0.55	0.74	0.63	0.42	0.55	0.055	0.38	0.76
6 wk	0.60	0.95	0.67	1.02	0.86	0.90	0.309	0.10	0.30
IgG (mg/mL)									
Initial	---------------------------------------------------------- 5.89 ----------------------------------------------------------								
3 wk	4.85	5.37	7.04	4.73	4.23	6.09	0.050	0.36	0.59
6 wk	4.39	7.64	7.03	6.85	6.40	7.41	0.311	0.07	0.10

SEM, standard error of the mean; IgA, immunoglobulin A; IgG, immunoglobulin G.

1) Least squares means of twelve observations per treatment.

2) CP16, corn-SBM-based diet (early weaning CP 16%/late weaning CP 15%); CP17, corn-SBM-based diet (early weaning CP 17%/late weaning CP 16%); CP18, corn-SBM-based diet (early weaning CP 18%/late weaning CP 17%); CP19, corn-SBM-based diet (early weaning CP 19%/late weaning CP 18%); CP20, corn-SBM-based diet (early weaning CP 20%/late weaning CP 19%); CP21, corn-SBM-based diet (early weaning CP 21%/late weaning CP 20%).

3) L, linear; Q, quadratic.

**Table 8:** Effects of different levels of dietary crude protein on nutrient digestibility in weaning pigs^1)^

Criteria	Treatment^2)^						SEM	p-value^3)^	
	
	CP16	CP17	CP18	CP19	CP20	CP21		L	Q
Nutrient digestibility (%)									
Dry matter	94.20	94.3	95.24	95.04	94.48	94.18	0.301	0.97	0.31
Crude protein	93.47	93.14	93.52	93.80	93.00	92.07	0.407	0.46	0.47
Crude ash	84.54	84.82	84.71	84.29	84.49	82.74	0.894	0.64	0.71
Crude fat	82.40	80.68	80.50	80.10	80.05	81.33	1.019	0.76	0.57
Nitrogen retention (g/d)									
N intake	12.15	12.22	13.24	14.04	14.56	15.07	-		
Fecal N	0.75	0.83	1.20	1.23	1.33	1.33	0.114	<0.01	0.64
Urinary N	3.03	2.93	3.50	4.18	4.63	5.13	0.029	<0.01	0.21
N retention^4)^	8.38	8.47	8.54	8.63	8.60	8.62	0.079	<0.01	0.50

SEM, standard error of the mean.

1) A total of 18 barrows (initial body weight, 8.41±0.371 kg).

2) CP16, corn-SBM-based diet (early weaning CP 16%/late weaning CP 15%); CP17, corn-SBM-based diet (early weaning CP 17%/late weaning CP 16%); CP18, corn-SBM-based diet (early weaning CP 18%/late weaning CP 17%); CP19, corn-SBM-based diet (early weaning CP 19%/late weaning CP 18%); CP20, corn-SBM-based diet (early weaning CP 20%/late weaning CP 19%); CP21, corn-SBM-based diet (early weaning CP 21%/late weaning CP 20%).

3) L, linear; Q, quadratic.

4) N retention = N intake (g) – fecal N (g) – urinary N (g).

**Table 9:** Effects of different levels of dietary crude protein on diarrhea incidence in weaning pigs^1)^

Criteria	Treatment^2)^						SEM	p-value^3)^	
	
	CP16	CP17	CP18	CP19	CP20	CP21		L	Q
Diarrhea score (%)^4)^									
0 to 3 wk	1.41	1.58	2.12	2.48	2.62	3.23	0.121	<0.01	0.52
3 to 6 wk	1.10	1.20	1.34	1.38	1.44	1.56	0.036	<0.01	0.72
0 to 6 wk	1.25	1.39	1.73	1.93	2.03	2.39	0.075	<0.01	0.76

SEM, standard error of the mean.

1) Least squares means for eight pigs per treatment.

2) CP16, corn-SBM-based diet (early weaning CP 16%/late weaning CP 15%); CP17, corn-SBM-based diet (early weaning CP 17%/late weaning CP 16%); CP18, corn-SBM-based diet (early weaning CP 18%/late weaning CP 17%); CP19, corn-SBM-based diet (early weaning CP 19%/late weaning CP 18%); CP20, corn-SBM-based diet (early weaning CP 20%/late weaning CP 19%); CP21, corn-SBM-based diet (early weaning CP 21%/late weaning CP 20%).

3) L, linear; Q, quadratic.

4) Diarrhea score: 0 = normal feces, 1 = moist feces, 2 = mild diarrhea, 3 = severe and watery diarrhea.

**Table 10:** Effects of different levels of dietary crude protein on odor emission in weaning pigs^1)^

Criteria	Treatment^2)^						SEM	p value^3)^	
	
	CP16	CP17	CP18	CP19	CP20	CP21		L	Q
Hydrogen sulfide (ppm)									
0 to 3 wk	1.75	2.00	2.00	2.25	2.00	2.25	0.041	<0.01	0.36
3 to 6 wk	1.50	1.83	1.83	2.33	2.33	2.33	0.079	<0.01	0.14
Ammonia (ppm)									
0 to 3 wk	5.36	5.36	6.45	6.91	7.64	8.64	0.286	<0.01	0.51
3 to 6 wk	5.27	5.36	6.36	6.91	7.55	8.18	0.259	<0.01	0.82
Amines (ppm)									
0 to 3 wk	50.91	51.82	53.64	70.91	77.27	81.82	3.080	<0.01	0.27
3 to 6 wk	45.45	47.27	51.82	76.36	77.00	77.27	3.511	<0.01	0.75

SEM, standard error of the mean.

1) A total of 18 barrows (initial body weight, 8.41±0.371 kg).

2) CP16: corn-SBM-based diet (early weaning CP 16%/late weaning CP 15%), CP17: corn-SBM-based diet (early weaning CP 17%/late weaning CP 16%), CP18: corn-SBM-based diet (early weaning CP 18%/late weaning CP 17%), CP19: corn-SBM-based diet (early weaning CP 19%/late weaning CP 18%), CP20: corn-SBM-based diet (early weaning CP 20%/late weaning CP 19%), CP21: corn-SBM-based diet (early weaning CP 21%/late weaning CP 20%).

3) L, linear; Q, quadratic.
